# Torsades de Pointes due to Excessive Marijuana Use in a Susceptible Patient

**DOI:** 10.1155/2021/6621496

**Published:** 2021-07-08

**Authors:** Vivek D. Shah, Adeba Mohammad, Shuktika Nandkeolyar, Liset Stoletniy, Tahmeed Contractor

**Affiliations:** ^1^Department of Internal Medicine, Loma Linda University Medical Center, CA, USA; ^2^Department of Internal Medicine, Division of Cardiology, Loma Linda University Medical Center, CA, USA

## Abstract

There are several recent reports of tetrahydrocannabinol vaping-related sudden cardiac arrest, and the mechanisms are unclear. We report a unique case of a 19-year-old female who suffered documented prolonged QTc leading to Torsades de pointes and cardiac arrest in the setting of frequent marijuana wax vaping. While she demonstrated normal baseline QTc measurements years earlier, she was found to have a genetic predisposition to QTc prolongation (genetic mutation, family history of prolonged QTc), suggesting that specific patient populations are at higher risk of these adverse events. The patient was acutely managed with isoproterenol to increase the heart rate and was discharged on nadolol after placement of an implantable cardioverter-defibrillator. Marijuana wax vaping and dabbing may cause fatal Torsades de pointes in susceptible patients, and further research is required to identify these patients a priori.

## 1. Introduction

In 2018, 3.9% of the global population from 15-64 years of age, or 192 million individuals, used tetrahydrocannabinol (THC), making it the most commonly used recreational drug [1]. This use has consistently increased since 2007 in young adults aged 18-25 with the advent of synthetic forms of cannabis [1]. These synthetic sources are up to 10 times more potent especially when consumed via wax vaping (dabbing), which involves the use of butane hash oil—made by extracting solely the hydrophobic components of marijuana plants. This form of cannabis is highly concentrated in THC (50%+) compared to the medicinal plant, which has a concentration of 4-8% [2]. With these shifts in the use and forms of THC come increasing evidence of associated adverse cardiac events including cardiac arrest, which may be related to prolongation of the QTc interval [3]. In this report, we discuss the case of a 19-year-old female who frequently used marijuana and suffered QTc prolongation-related ventricular fibrillation (VF) arrest at home followed by recurrent Torsades de pointes (Tdp) during hospitalization. Unique to her case is a normal QTc interval in the past, genetic mutation for long QT syndrome type 2 (LQTS2), and family history of sudden cardiac arrest as well as QTc prolongation. This case highlights the fact that certain individuals may be at risk of fatal arrhythmias with the use of recreational marijuana that is otherwise considered safe and legal in many states in the USA.

## 2. Case Presentation

### 2.1. Patient Information

**Figure 1:**
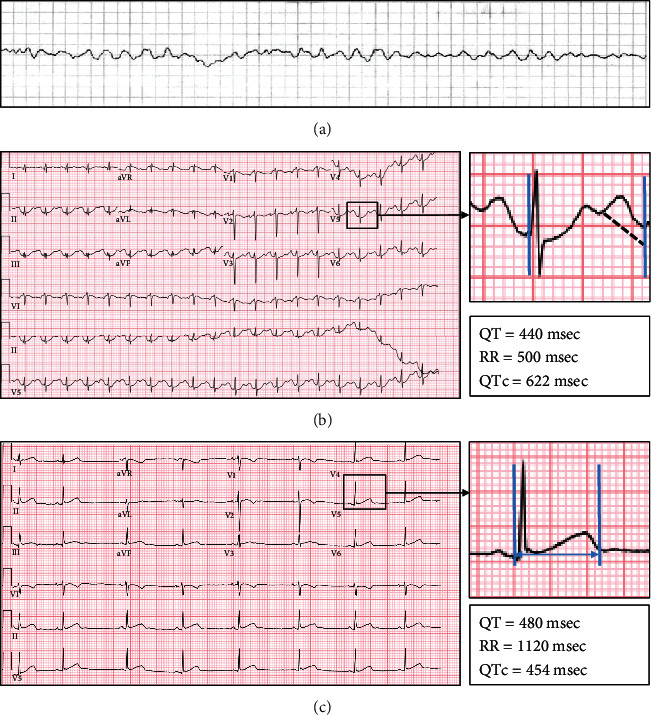
(a) Ventricular fibrillation detected by emergency medical services requiring defibrillation; (b) QTc measured in ECG obtained after correction of electrolyte abnormalities (measured by U-wave avoidance technique and calculated by the Bazett Formula: $\text{QTc}=\text{QT}/\sqrt{\text{RR}}$); (c) measurement of QTc from baseline ECG done several years prior.

A 19-year-old female with a family history of prolonged QTc and social history of frequent use of marijuana through dabbing had a witnessed episode of loss of consciousness. The family performed cardiopulmonary resuscitation for 20 minutes until emergency medical services arrived. The initial rhythm was VF (Figure 1(a)), and she was defibrillated with return of spontaneous circulation. She was intubated at the scene and transferred to our hospital. A detailed assessment of her family history revealed sudden cardiac arrest in a 28-year-old maternal cousin. A maternal aunt died of cardiac arrest at age 65 years. The patient's mother and sister both had a history of prolonged QTc diagnosed on electrocardiogram (ECG) by each of their primary care physicians with no further work-up performed.

### 2.2. Clinical Findings

Upon arrival to the hospital, the initial physical exam was notable for a respiratory rate of 24 and heart rate of 114. She had mechanical breath sounds bilaterally.

### 2.3. Diagnostic Assessment

Our differential diagnosis for Tdp-related cardiac arrest included QTc prolongation (either from inherited arrhythmia syndromes or secondary causes such as drug ingestion and electrolyte derangements), Brugada syndrome, catecholaminergic polymorphic ventricular tachycardia (CPVT), and structural heart diseases such as arrhythmogenic right ventricular dysplasia (ARVD) or hypertrophic cardiomyopathy.

Initial labs demonstrated metabolic acidosis with pH 7.28; all electrolytes were within normal limits with a magnesium level of 1.2 (reference 0.7-1.1 mmol/L) and a potassium level of 3.7 (reference 3.5-5.0 mmol/L). Cardiac enzymes were obtained, revealing mildly elevated troponin T levels at 0.04 ng/mL (normal ≤ 0.03 ng/mL), which increased to a maximum of 0.96 ng/mL and then normalized. Toxicology work-up was negative for acetaminophen, salicylate, and prescription drug toxicity. Urine drug screen revealed cannabinoid use. ECG was obtained after correction of electrolyte abnormalities and showed prolonged QTc of 622 msec (Figure 1(b)). Notably, previous ECG on record from 5 years prior was normal with a QTc of 454 msec (Figure 1(c)). Initial echocardiogram revealed left ventricular ejection fraction (LVEF) of 25-30%.

### 2.4. Therapeutic Intervention

Hypothermia protocol was initiated, and the patient was admitted to the intensive care unit. Initial management included (1) intravenous calcium gluconate and magnesium for prolonged QTc, (2) propofol for sedation, and (3) amiodarone drip for prevention of recurrent ventricular arrhythmias.

We provided calcium carbonate and magnesium as treatments for this patient's arrhythmia before initial labs were available to ensure stabilization of the cardiac membranes and since hypocalcemia and hypomagnesemia are known risk factors for prolonged QTc and TdP [4, 5].

**Figure 2:**
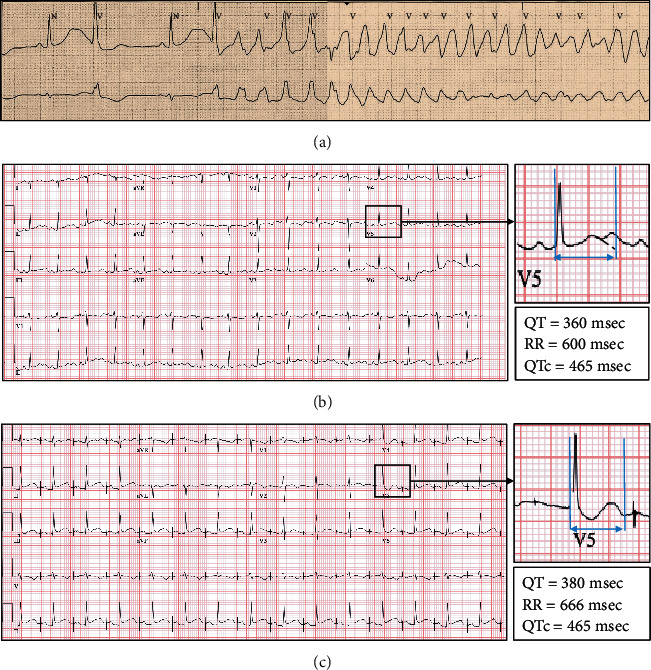
(a) Telemetry strip showing bradycardia with prolonged QTc and R-on-T premature ventricular contraction initiating Torsades de pointes; (b) ECG after starting isoproterenol drip with QTc reduction to 465 msec. (c) ECG showing atrial pacing after placement of dual-chamber ICD, with QTc of 465 msec, prior to starting nadolol.

After admission, she had two episodes of Tdp, requiring cardioversion. Telemetry demonstrated persistently prolonged QTc intervals and bradycardia with a subsequent R-on-T premature ventricular complex initiating these episodes (Figure 2(a)). Amiodarone drip was thus discontinued, and the patient was started on an isoproterenol drip with a decrease in QTc to 465 msec and resolution of ventricular arrhythmias (Figure 2(b)), and the patient was extubated in three days.

Cardiac magnetic resonance imaging (MRI) with delayed gadolinium was performed, which did not demonstrate any scar but did demonstrate a normalized LVEF, indicating that the initial echocardiographic changes were likely related to the cardiac arrest. While the prolonged QTc and the resultant TdP could have been “reversible” from marijuana use, the propensity of the patient to develop future episodes with other QTc-prolonging medications/triggers, especially with significant family history, was discussed. In a shared decision-making process with the patient and her family, it was decided to proceed with dual-chamber implantable cardioverter-defibrillator (ICD). At the time of discharge, she was atrially paced at 90 BPM (Figure 2(c)) and started on nadolol 40 mg twice daily.

The patient underwent genetic testing for long QT syndromes, which revealed a variant of unknown significance in KCNH2 (exon 10 c.2407_2409del (p.Asp803del)). Gene KCNH2 (also known as hERG) has been implicated in LQTS2; however, the specific variant seen in this patient has yet to be reported as associated with risk for LQTS. She was also noted to have mutations in PKP2, which is associated with ARVD, and TRDN, which is associated with CPVT. The normal MRI and QTc prolongation-related VF episodes did not support a clinical diagnosis of ARVD or CPVT.

### 2.5. Follow-Up and Outcomes

At the three-month follow-up, the patient had no further episodes of Tdp, and interrogation of ICD revealed no recurrence of ventricular arrhythmias. Her atrial pacing rate was dropped to 70 beats per minute, with QTc found at 463 msec. Echocardiogram performed at the three-month follow-up reveals an ejection fraction of 65%.

## 3. Discussion

**Figure 3:**
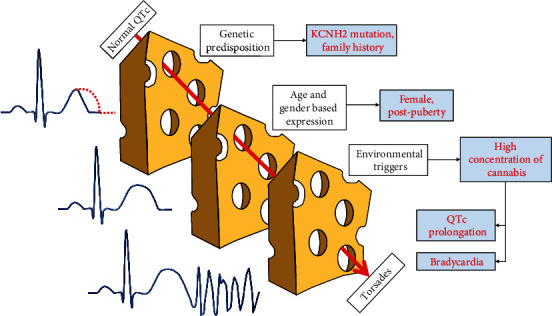
Representation of “the perfect storm” that led up to Tdp and cardiac arrest. Normal QTc several years ago was likely secondary to lack of age/gender-based expression. However, when postpubertal and in the presence of a potent environmental trigger, she developed QTc prolongation and bradycardia resulting in Tdp and cardiac arrest.

THC has been associated with different types of ventricular arrhythmias, including VF from QTc prolongation or Brugada syndrome and monomorphic VT attributed to ischemia [3, 6, 7]. This case highlights the importance of pharmacogenomics in the pathophysiology of arrhythmia associated with the relatively common practice of THC dabbing. In this patient with a normal QTc interval as a child, the combination of (1) genetic predisposition (family history, genetic mutation), (2) being a postpubertal female, and (3) consumption of high-concentration THC products provided the “perfect storm” for Tdp [8] (Figure 3). THC can cause Tdp via 2 mechanisms: (1) chronic THC results in increased parasympathetic and reduced sympathetic activity, with resultant bradycardia that predisposes to further prolongation of QTc and early after depolarizations [9], and (2) use of marijuana has been associated with long QTc via inhibition of the delayed rectifier potassium channel (coded for by hERG gene) [10, 11], through a mechanism similar to LQTS2 [12]. Genetic testing in this patient revealed a mutation in genes coding the same ion channels associated with THC-induced QTc prolongation.

The management of THC vaping or dabbing-related Tdp remains unclear. As in other cases of prolonged QTc-mediated TdP, increasing the heart rate with isoproterenol may provide a temporary solution. As a nonspecific *β*_1_/*β*_2_-adrenoceptor agonist, isoproterenol increases the heart rate, thus shortening the QTc interval and refractory period [5]. Long-term management requires a shared process of decision-making regarding consideration of ICD, in conjunction with pharmacologic therapies. Beta blockers, such as nadolol, may reduce recurrence of ventricular arrhythmias, with the postulated mechanism being a combination of reduction in dispersion of repolarization across the myocardium, decrease in after depolarizations, and in some cases a shortened QTc [13]. In addition, nadolol blocks late Na-currents (i.e., *I*_Na_ that continue to flow during the action potential plateau) that then shortens the repolarization phase [14]. In the absence of any other obvious triggers, we also recommend such patients abstain from further use of marijuana products and any other potential QTc-prolonging products including certain medications.

## 4. Conclusions

There are an increasing number of cases reporting sudden cardiac arrest related to THC use, and the mechanisms are unclear (3). In the setting of genetic propensity for QTc prolongation, THC dabbing, which results in high concentrations of the compound, may be a potent trigger of TdP due to a combination of sympathetic suppression by a high concentration of THC, and QTc prolongation, even in the setting of a baseline normal QTc interval. Genetic testing may assist in identifying patients at the highest risk of arrhythmia, initially screened with appropriate family history. Acute treatment involves temporary shortening of QTc interval through nonspecific beta agonists such as isoproterenol. Long-term management may involve the use of nonspecific beta blockers, shared decision-making to determine appropriateness of ICD, and avoidance of QTc-prolonging triggers.

## 5. Patient Perspective

The patient had limited recollection of her hospitalization course; however, she has expressed her gratitude regarding her hospital and postdischarge care. She reports that after her experience, she has stopped using cannabis and has made drastic changes to her lifestyle including exercise and diet changes. As a result of her hospitalization, her sister has stopped using cannabis and has inspired her peers to also refrain from using it. Her mother and sister visited their primary care physicians and were found to have prolonged QTc on screening ECGs. They were instructed to avoid QT-prolonging drugs but have not performed genetic testing yet.

## Abbreviations

THC: Tetrahydrocannabinol
VF: Ventricular fibrillation
Tdp: Torsades de pointes
LQTS2: Long QT syndrome 2
ECG: Electrocardiogram
CPVT: Catecholaminergic polymorphic ventricular tachycardia
ARVD: Arrhythmogenic right ventricular dysplasia
ICD: Implantable cardioverter-defibrillator
LVEF: Left ventricular ejection fraction
MRI: Magnetic resonance imaging.

## References

[1] UNODC World Drug Report Drug Use and Health Consequences. http://www.unodc.org/wdr2020.

[2] Al-Zouabi I., Stogner J. M., Miller B. L., Lane E. S. (2018). Butane hash oil and dabbing: insights into use, amateur production techniques, and potential harm mitigation. *Substance Abuse and Rehabilitation*.

[3] Ahmed T., Khan A., See V. Y., Robinson S. (2020). Cardiac arrest associated with synthetic cannabinoid use and acquired prolonged QTc interval: a case report and review of literature. *HeartRhythm Case Reports*.

[4] Eryol N. K., Çolak R., Özdoğru (2003). Effects of calcium treatment on QT interval and QT dispersion in hypocalcemia. *The American Journal of Cardiology*.

[5] Thomas S. H., Behr E. R. (2016). Pharmacological treatment of acquired QT prolongation and torsades de pointes. *British Journal of Clinical Pharmacology*.

[6] Sampat P. J., Riaz S., Bisen M., Carhart R. (2020). An Unusual Case of Ventricular Tachycardia in a Young Patient Associated with Cannabis Use. *Case reports in Cardiology*.

[7] Kariyanna P., Wengrofsky P., Jayarangaiah A. (2019). Marijuana and cardiac arrhythmias: a scoping study. *International journal of clinical research & trials*.

[8] Sedlak T., Shufelt C., Iribarren C., CNB M. (2012). Sex hormones and the QT interval: a review. *Journal of Women's Health*.

[9] Goyal H., Awad H. H., Ghali J. K. (2017). Role of cannabis in cardiovascular disorders. *Journal of Thoracic Disease*.

[10] Ramphul K., Joynauth J. (2019). Cardiac arrhythmias among teenagers using Cannabis in the United States. *The American Journal of Cardiology*.

[11] Yun J., Yoon K. S., Lee T. H. (2016). Synthetic cannabinoid, JWH-030, induces QT prolongation through hERG channel inhibition. *Toxicology Research*.

[12] Shimizu W., Moss A. J., Wilde A. A. (2009). Genotype-phenotype aspects of type 2 long QT syndrome. *Journal of the American College of Cardiology*.

[13] Shah M., Carter C. (2008). Long QT syndrome: a therapeutic challenge. *Annals of Pediatric Cardiology*.

[14] Besana A., Wang D. W., George A. L., Schwartz P. J. (2012). Nadolol block of Nav1.5 does not explain its efficacy in the long QT syndrome. *Journal of Cardiovascular Pharmacology*.

